# Bond Strength of Nanocomposite Hard Liner to CAD-CAM Milled, 3D Printed, and Conventionally Fabricated Denture Base Resins

**DOI:** 10.3390/dj12090275

**Published:** 2024-08-23

**Authors:** Zainab Albazroun, Atheer Alabdullatif, Sarah Aldehaileb, Ferdoos Alhalimi, Faris A. Alshahrani, Soban Q. Khan, Shaimaa M. Fouda, Hamad S. AlRumaih, Mohammed M. Gad

**Affiliations:** 1 College of Dentistry, Imam Abdulrahman Bin Faisal University, P.O. Box 1982, Dammam 31441, Saudi Arabia; 2190003488@iau.edu.sa (Z.A.); 2200002242@iau.edu.sa (A.A.); 2190004196@iau.edu.sa (S.A.); 2180006408@iau.edu.sa (F.A.); 2 Department of Substitutive Dental Sciences, College of Dentistry, Imam Abdulrahman Bin Faisal University, P.O. Box 1982, Dammam 31441, Saudi Arabia; faalshahrani@iau.edu.sa (F.A.A.); hsalrumaih@iau.edu.sa (H.S.A.); 3 Department of Dental Education, College of Dentistry, Imam Abdulrahman Bin Faisal University, P.O. Box 1982, Dammam 31441, Saudi Arabia; sqkhan@iau.edu.sa

**Keywords:** CAD-CAM, complete denture, bond strength, nanoparticles, denture liners

## Abstract

Background: To investigate the effect of zirconium dioxide nanoparticles (ZrO_2_NPs) on the shear bond strength (SBS) of hard denture lines bonded to different denture base resins. Methods: Five different denture bases were used in this study: conventional heat-cured resin, IvoCad, AvaDent, NextDent, and FormLabs, in acrylic specimens of 10 × 10 × 2.5 mm^3^ (N = 150, n = 10). Specimens were centered at the bottom of a silicon mold to create an auto-polymerized holder. Three major groups of reline material were used: no ZrO_2_NPs (control), 2 wt.%, and 4 wt.% ZrO_2_NPs. Reline was bonded to the resin surface using a customized jig. After polymerization, specimens were stored in distilled water, and 5000 thermal cycles were performed. Each specimen was fixed to an Instron machine, and SBS was tested using a blade loaded (1 mm/min) at the resin interface until failure. Data was collected and analyzed using two-way ANOVA and post hoc Tukey test (α = 0.05). Results: AvaDent showed the highest SBS when compared with other denture base materials (*p* < 0.001) except for IvoCad. The addition of ZrO_2_NPs significantly decreased the SBS of AvaDent (*p* = 0.003) and IvoCad (*p* = 0.001), while heat polymerized resin, Formlabs, and NextDent showed no significant change in SBS (*p* > 0.05). Conclusion: CAD-CAM milled denture base resin showed higher SBS with pure denture reline. The addition of ZrO_2_NPs decreased the SBS of reline with CAD-CAM milled denture base resins but did not change bond strength with 3D printed and conventional denture base resins.

## 1. Introduction

The population of edentulous patients has been growing continuously, leading to dramatically increased demand for prosthodontic therapies [1]. Complete dentures are used to treat edentulous patients, restoring function and appearance. However, with denture use, occlusal forces are transmitted to the underlying bony ridge, leading to gradual bone resorption and subsequent decreased denture retention and stability [1,2]. Accordingly, relining is necessary to improve denture fit to the underlying supporting tissue [3]. 

Denture relining is performed to resurface the intaglio surface of the denture when minimal bone changes occur while the denture is relatively in good condition. Thus, denture relining is indicated to avoid fabricating a new denture [3]. Relining can be done directly intraorally with chairside materials. Chairside denture relining materials are classed as either hard or soft liners. Hard reline resins are used to enhance the stability and retention of ill-fitting dentures and as interim liners in the case of immediate dentures. There are several available types, including auto-polymerized and visible light-polymerized polymers [4]. Soft reline materials are a class of resilient materials used for the rehabilitation of edentulous patients suffering from pain from traumatized oral mucosa [5,6]. They should possess sufficient tear strength, dimensional stability, color stability, and viscoelastic properties, in addition to adequate bond strength with denture base resins [7]. Tensile and shear bond strengths are affected by the chemical composition of the reline and denture base materials. A weak bond between them can result in increased bacterial adhesion, discoloration, separation of the lining material, and decreased strength of the relined denture base [3,8].

The combination of computer-aided design and computer-aided manufacturing (CAD-CAM) has been increasingly used for the fabrication of dental prostheses. It is important to study the bonding between denture liners and CAD-CAM denture base materials to ensure proper clinical application. Nevertheless, few studies have investigated the nature of this bond [9]. A primary investigation into the bonding of liners to different denture base resins reported that auto-polymerized resins exhibited the highest potential for bonding with different types of resilient liners; CAD-CAM denture base resins exhibited the lowest tensile bond strength [9]. A previous study by Al Taweel et al. [10] found comparable tensile bond strength in conventional and CAD-CAM acrylic resins applied to soft denture liners. 

Multiple nanoparticles (NPs) are added to denture liners, mainly to enhance the antimicrobial activities of nanocomposite liners; these include fluorescent carbon [7], copper oxide [11], and magnesium oxide [12]. Other studies [7,13] have reported the effects of NPs on denture liners, finding antioxidant and antibacterial features without changes to mechanical properties [7].

Recently, the addition of zirconium dioxide nanoparticles (ZrO_2_NPs) to hard liners was tested, and its antifungal activities were reported as a potential treatment for denture stomatitis [14]. However, the effect of adding ZrO_2_NPs on the mechanical behavior of denture liners has not been investigated. Studies investigating the effects of adding ZrO_2_NPs have done so at specific concentrations: 0.5 wt%, 1 wt%, 3 wt%, and 5 wt% [15,16]. Therefore, this study aimed to investigate the effect of ZrO_2_NPs at different concentrations (2 wt% and 4 wt%) on the shear bond strength (SBS) of denture liners bonded to different base resins. The null hypothesis is that the addition of ZrO_2_NPs has no effect on the SBS of denture liners to base resins.

## 2. Materials and Methods

Power analysis was performed to calculate the sample size. For this purpose, a study published by Gad et al. [17] was used to extract the mean and standard deviation. The power was set at 80% and the confidence interval at 95%. The calculated sample size for the study was 10 samples per group. Therefore, a total of 150 specimens were used to test SBS. Five different denture base materials (two CAD-CAM-milled, IvoCad (IVO) and AvaDent (AVA); two 3D printed resin, FormLabs (FL) and NextDent (ND); and one heat-polymerized resin (HP)) were used in this study with one hard denture reline (Table 1).

**Figure 1:**
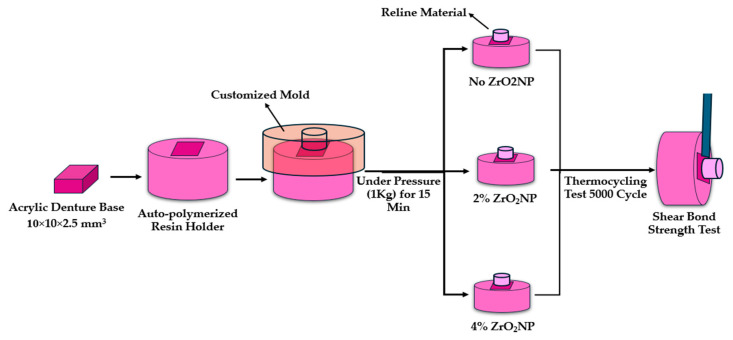
Illustration of the specimen preparation and repair procedure standardization.

Acrylic denture bases were prepared in the dimensions 10 × 10 × 2.5 mm^3^ according to manufacturers’ recommendations. The polishing procedure was standardized for all specimens and performed by one investigator. Each specimen was fixed in an acrylic holder using a silicone jig. Denture base specimens had been positioned and centered at the bottom of the jig and then filled with the auto-polymerized resin. After polymerization, resin flashes were eliminated, demonstrating the clarity of the denture base resin specimen (Figure 1).

ZrO_2_NPs (<100 nm in size, 99.9% purity) was weighed and added to hard chairside denture reline material (GC AMERICA INC. Alsip, IL 60803 U.S.A) in two concentrations (2% and 4% wt.). ZrO_2_NPs was added to reline powder and thoroughly mixed until a homogenous mixture was obtained, according to methods detailed in a previous study [18]. The specimens of each group were divided into three subgroups: control without ZrO_2_NPs and two experimental groups (2%- and 4%- ZrO_2_NPs).

At the center of the prepared denture base specimen, a metal cylinder (4 mm diameter × 6 mm length) was fixed within the holder. A customized mold was prepared, into which each specimen was fixed. The hard liner was then mixed and packed in the space created by the cylinder and covered with a glass slap under pressure (1 kg) for 15 min. After polymerization, the specimens were stored in distilled water (37 °C) for two days and then subjected to 5000 thermal cycles (5 °C to 55 °C with a 30-s dwell time) within a thermal cycling device (Thermocycler, THE-1100/THE-1200, SD Mechatronik GMBH Miesbacher, Westerham, Germany).

SBS was evaluated by a universal testing device (Instron, Instron Corp., Norwood, MA, USA). Specimen was placed and secured in a specially designed machine jig using a chisel (knife-edge shear type), and the load was delivered as closely as possible to the resin interface with a cross-head speed of 1 mm/min until specimen failure. The formula (MPa) = F/A, where F is the force (N) and A is the bonding area, was used to calculate the SBS. 

After SBS assessment, the de-bonded specimen surface was prepared and gold-sputtered for scanning electron microscope (SEM) analysis (TESCAN VEGA 3, Brno, Czech Republic; operated at 20 kV). The specimen surface was examined by one investigator using dental loops with 3x magnification for fracture type and mode of failure analysis. Failure mode was classified as adhesive, cohesive, or mixed according to previously reported criteria [19].

Normality of the data was checked using the Shapiro-Wilk test and was confirmed to be normally distributed. Parametric tests were then used for inferential analysis. One-way ANOVA was used to study the relationship between a continuous and a categorical variable with more than two categories. Tukey’s post hoc test was used for pair-wise comparisons. Two-way ANOVA was used to study the combined effect of two categorical variables on the continuous variable. All *p*-values less than 0.05 were considered statistically significant.

## 3. Results

### 3.1. SBS

Table 2 shows the mean, standard deviation (SD), and significance between groups concerning nanoparticle-modified reline and denture base resin. For unmodified groups, AVA showed the highest bond strength when compared with all other denture base materials (*p* = 0.000) except with IVO there is no statistical significant difference. The addition of ZrO_2_NPs significantly decreased the SBS of AVA (*p* = 0.003) and IVO (*p* = 0.001), while HP, FL, and ND were not significantly altered in SBS (*p* > 0.05).

Overall, ZrO_2_NPs treatment at 2% showed a statistically significant difference in SBS (*p* = 0.033), while at 4% produced no significant change (*p* = 0.372). When comparing both concentrations per resin, no significant differences were found (*p* > 0.05) except for IVO, which showed a significant difference between 2% and 4% (*p* = 0.001).

Two-way ANOVA was used to study the combined effect of concentration and materials on the tested property (Table 3). It was found that the combined effect of material and concentration was statistically significant (*p* < 0.001).

### 3.2. Type of Failure

**Figure 2:**
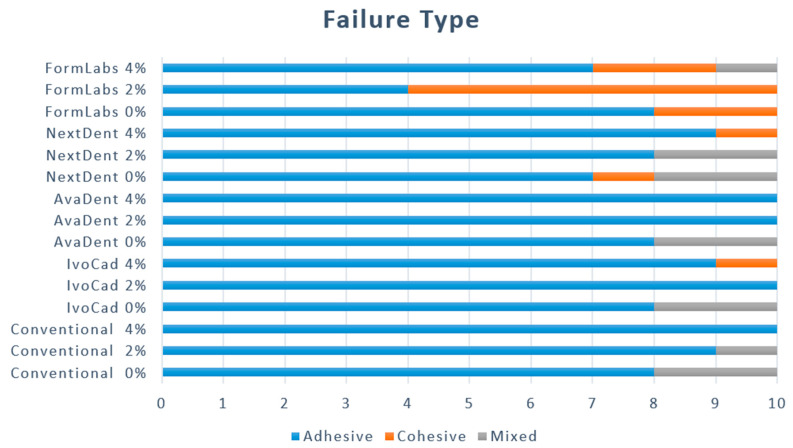
Mode of failure variation between tested groups.

**Figure 3:**
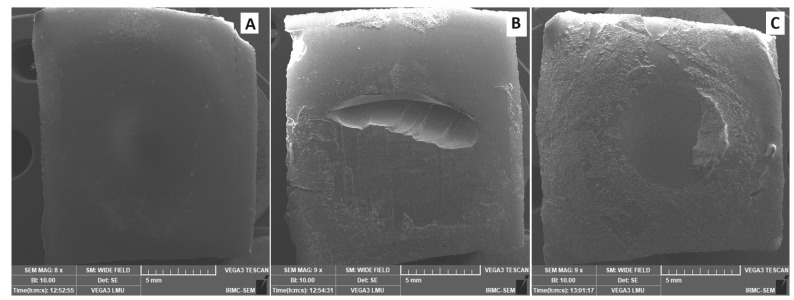
SEM representative image of different failure modes. (**A**) Adhesive type, (**B**) Cohesive type, and (**C**) Mixed type.

The failure mode results (Figure 2) show the behavior of specimens after de-bonding in terms of adhesive, cohesive, or mixed failure. All groups demonstrated the adhesive type of fracture, except FL with 2% ZrO_2_NPs, which displayed a cohesive failure (within denture base resin) as the dominant type. According to SEM representative images, complete de-bonding of reline resin with a smooth background of denture base resin represents adhesive type (Figure 3A). While the cohesive failure within the resin represents the void at the center of the de-bonded area (Figure 3B). Figure 3C exhibited mixed fracture type as some reline materials remained on the denture base surface.

## 4. Discussion

In this in vitro study, the SBS of ZrO_2_NPs-modified hard liner bound to conventionally and CAD-CAM fabricated denture base resins was tested. Adding ZrO_2_NPs to denture liner significantly decreased the SBS when bound to CAD-CAM milled groups, while no significant change was observed with conventional or 3D-printed groups. Therefore, the null hypothesis was partially rejected.

Evaluation of the bond strength of relining material is usually determined by measuring the tensile, shear, and peel bond strengths [20,21]. McCabe et al. [22] recommend peel and tensile methods to test the bonding and de-bonding characteristics of soft liners. The peel test is supposed to replicate the horizontal part of the masticatory forces that could result in the denture lateral movement and the liner being stripped at the denture flanges [23]. Tensile bond strength contains part of the shear force failure, which is why it’s advised to use it with different adhesive systems [24,25,26]. Shear load is applied at the denture/reline interface and is superior to tensile load because it is subject to stresses in different directions during chewing cycles. Additionally, this test has been implemented in previous studies to measure bond strengths between denture base resins and reline materials [10,25,26]. Accordingly, the shear bond test was implemented in this study.

Enhancing the quality and clinical behavior of printed complete dentures requires an understanding of how printing conditions affect the properties of 3D-printed base resin [27]. Yacob et al. [28] demonstrated a low affinity for microbial adherence using a 3D-printed denture base material that was constructed with a 0-degree orientation. Yan et al. [29] also recommend a 0-degree build angle, as it provides the most favorable base surface characteristics, including smooth surface and high hydrophilicity, and demonstrates the best trueness and precision. Based on these reports, a 0-degree orientation for printing was used in this study.

Adequate bonding between the denture base and relining material is required for long-term clinical use and to avoid de-bonding of liners [30]. The bond strength of denture liners is affected by several factors, including the chemical structure of the denture base and relining materials, the thickness of the lining material, and thermal stress [9,24,31]. The dental prosthesis is exposed to significant temperature changes during clinical usage due to the consumption of meals and beverages at different temperatures. Thermal cycling also provides specimen hydration by immersion in water at various temperatures, simulating the oral environment [32]. Water sorption is the most common reason for weakened bond strength, which is due to the swelling of resins and the effects of bonding at the interface [33]. Water uptake increases at higher temperatures, which can exaggerate the adverse water sorption effect [34]. Absorbed water molecules have a plasticizing function that can infiltrate the junction of the denture base and the reline, weakening the bond [33,35]. Due to these various effects, temperature cycling is an important test for the bond strength of hard reline and denture base materials [35], simulating aging and intra-oral conditions. All prepared specimens were exposed to 5000 thermal cycles, simulating 6 months of clinical use [34].

Assessing the type of material failure is important for evaluating tensile bond strength test results. Adhesive failure shows that the bond between the liner molecule and base resin is weaker than the bond between the relining material, whereas cohesive failure shows that the strength of the material is lower than the bond strength [22,26]. Consistent with previous studies, adhesive failure was the dominant type, confirming that the bond at the resin/reline interface is the weakest point while also demonstrating the strong bond of the liner itself after NP addition [26,36].

Previous research has shown that the more comparable the chemical structure of the denture base and the relining materials, the stronger the bonding [25,37]. However, because chemical structure is an inherent characteristic of materials, dentists cannot manipulate it in clinical situations. The ability to create a strong binding between the substrate and repair resin is essential for a successful treatment, as well as the compatibility of both the material to be repaired and the repair material itself. This was demonstrated by Viotto et al. [38], who discovered an increasing cohesive failure and weak bond strength as a result of substrate and repair material incompatibilities. It is unknown at this time [39] if repair resins and 3D-printed prostheses are compatible. The durability of the repair junction is influenced by the substrate and repair resin that form strong connections. This is determined by the quantity of unconverted C=C bonds present at the junction [39]. Regretfully, compared to conventional resin, C=C bonds are less common in acrylic resin that is 3D printed [40].

The minimum accepted bond strength for hard denture liners is 4–6 MPa [3,41,42]. A weak bond between the reline material and the denture base leads to bacterial accumulation, discoloration, poor oral hygiene, and, eventually, reline material separation [9]. Several factors affect the bond strength of relining material, including the chemical composition of the bonded materials, liner thickness, nature of the adhesive, tear strength, and thermal stresses [35,43]. Milled resin materials showed the highest SBS among all those tested. This agrees with a previous study [44] comparing injected, milled, and 3D-printed resins bonded to different liners. Awad et al. [44] demonstrated that the milled denture base showed the highest bond strength values.

On the other side, 3D-printed resins showed the lowest bond strength of those tested. This is consistent with previous studies that compared different denture base resins bonded to different hard relines [44,45]. Low bond strength could be caused by the high residual monomer content in both 3D-printed resin and auto-polymerizing resin, which would negatively impact the mechanical properties [41]. The porous surface of 3D-printed resin could also be a reason for the weak bond with the liner material; diffusion of residual monomers into these pores prevents polymer network formation and penetration [26,46]. Consistent with the present results, Gad et al. [17] found lower repair bond strength of 3D-printed resin than conventional and milled resins.

The method of bonding between conventional denture base resins (PMMA) and the chairside liner material starts with the swelling of the base resin after application of the monomer at the intaglio surface of the denture base. Afterwards, the relining material is added, and the monomers penetrate and diffuse; an interpenetrating polymer network (IPN) is produced through polymerization [45]. Bond strength is affected by the thickness of the IPN, which is determined by the swelling of the PMMA and the diffusion of the monomer [45].

The cause of the apparent decrease in bonding strength at greater ZrO_2_NPs concentrations is unclear. At high concentrations, ZrO_2_NPs agglomeration occurred with cluster formation; this results in the presence of NPs at the reline/resin interface which could affect the bond strength. This is consistent with a previous study [47] that investigated the bond strength between acrylic teeth and denture bases containing ZrO_2_NPs and found that bond strength decreased as ZrO_2_NPs concentration increased. Another potential factor is the surface properties of substrates, bonding mechanism, and expected change at the interface when relined using nanocomposite liner material. This would be supported by adhesive failure being the most common mode.

In general, the addition of ZrO_2_NPs to hard liner did not affect the bond strength with 3D-printed denture base resins. This may be due to the aforementioned surface properties of 3D-printed resin derived from the nature of the printing. Reline procedures were conducted according to the manufacturer’s recommendation only, without surface treatment. Surface treatment might have improved the bond strength between the hard liner and the tested denture base resins [2,31,48]. This finding is supported by the primarily adhesive failures of all tested groups, demonstrating weak bonds at the base/reline interface. However, cohesive failure was frequently reported with 2%FL, including with 3D-printed resins. This finding further indicates the unclear bonding mechanism at the resin reline interface, with or without nanoparticle addition.

Previous studies [16,19,49] have investigated the addition of varying concentrations of ZrO_2_NPs (1–7.5 wt.%). Qaw et al. [19] reported that up to a concentration of 5%, the repair bond strength increased, while above 5%, there was no significant change. In an earlier study by Yasser et al. [49], the addition of ZrO_2_NPs to the soft denture lining provided an antifungal property. SBS also increased after the incorporation of ZrO_2_NPs at a 1.5% concentration into the soft liner, leading to the recommendation to increase the use of ZrO_2_NPs [43]. Al-Tu’ma et al. [50] report a significant drop in *C. albicans* colony-forming units with soft liners reinforced with 2% ZrO_2_NPs. Therefore, the effects of two concentrations (2% and 4%) on SBS were investigated in the present study [19,49,50]. Concerning the effect of concentration, there was no significant change in SBS between denture base materials except for milled resins. With both concentrations, SBS decreased, showing the lowest value with IVO at 4% among milled groups. As NP concentration increased, agglomeration and cluster effects appeared, which are the reasons for the loss of bond strength reported in previous studies Abdulrazzaq Naji et al. [16] found that adding ZrO_2_NPs decreased the bond strength between artificial teeth and acrylic denture base resin. Qaw et al. [19] suggest ZrO_2_NPs may increase the repair bond strength of acrylic resin but found a slight decrease in SBS above 5%.

All SBS values were higher than the clinically recommended value of 4–6 MPa; therefore, using the ZrO_2_NPs/liner mixture is recommended due to its antifungal properties. According to the findings of the present study, ZrO_2_NPs could be considered when selecting nanocomposite denture liners. Additionally, denture base resin type has a positive impact on SBS, so denture base selection is another factor to consider. The difference in denture base compositions and reline material could be an obvious reason for the variation in outcomes. Therefore, these results must be treated with caution until further investigations are conducted. According to the present results, the type of denture base affected the SBS of the hard liner. 3D-printed and conventional resins can be relined with hard liners, including 2% and 4% ZrO_2_NPs as antifungal agents, without altering the SBS. While for milled resins, the addition of ZrO_2_NPs should be considered with caution due to the decreased SBS, its value was still higher and similar to those of 3D-printed and conventional resins at 2% and 4%, respectively.

The strength of the present study was in testing different denture base resins after exposure to artificial aging. However, as an in vitro study, it is limited by the lack of the entire intraoral environment, such as occlusal forces, saliva pH variations, and intraoral flora. Furthermore, the tested specimens did not resemble the denture configurations, and the specimens were not subjected to aging before the relining procedures. For 3D-printed resins, printing orientations affect the material properties, and only a 0-degree orientation was investigated. Finally, only one reline material and two ZrO_2_NPs concentrations were evaluated. Consequently, further studies are required to test SBS with various reline materials in conditions representing the intraoral environment. In addition, testing different concentrations of ZrO_2_NPs is important to determine the most appropriate application. Moreover, further investigations of different liners with nanoparticles and different resin substrate surface treatments are recommended. The research model currently explores shear bond strength in a unidirectional manner, which may not fully represent clinical conditions. Incorporating finite element analysis to simulate multidirectional stress could increase clinical relevance.

## 5. Conclusions

Milled denture base resins showed the highest SBS with pure denture liners. The addition of ZrO_2_NPs to the denture liner did not change the SBS of conventional and 3D-printed resins, while it decreased that of milled base resins. However, the SBS values for all groups were above the minimum acceptable value, therefore, ZrO_2_NPs addition to hard reline is recommended and could be used for the relining of HP, milled, and 3D-printed denture base resins due to its antifungal activities.

## Figures and Tables

**Table 1:** Specifications of materials used and different fabrication technologies.

Materials (Brand Name)	Composition	Specimens Fabrication Method
Heat polymerized resin (HP) (Major Base.20, Major Prodotti Dentari Spa, Momcalieri, Italy)	Powder: Polymer (PMMA) þ initiator (benzoyl peroxide [BPO]) (0.5%) þ pigments (salts of cadmium or iron or organic dyes) Liquid: Monomer (MMA) þ cross-linking agent (Ethylene glycol dimethacrylate [EGDMA] 10%) þ inhibitor (hydroquinone)	Specimens fabricated conventionally via heat polymerization (heat water bath with increased temperature up to 74 °C for 90-min. followed by 100 °C for 30 min.
IvoCad (IvoCad, Ivoclar Vivadent, Schaan, Liechtenstein)	Prepolymerized PMMA discs 50–100% methyl methacrylate 2.5–10% 1,4-butanediol dimethacrylate	The pre-polymerized discs were mounted on a cutting saw ((Isomet 5000 Linear Precision Saw, Buehler Ltd., Bluff, IL, USA) to cut the specimens to the required dimensions using a diamond saw
AvaDent (AvaDent Digital Dental Solutions, Scottsdale, AZ, USA)	Prepolymerized PMMA (PMMA 99.5%, pigments < 1.0%)	
NextDent Denture 3D+ NextDent B.V., Soesterberg, The Netherlands	Ester-based monomer; Bisacylphosphine oxide (BAPO) phenylbis (2,4,6- trimethylbenzoyl)-phosphine oxide (Omnirad 819)	NextDent 5100 3D printer was used to print specimens with 50 µm Printing layer thickness and 0-degree printing orientation. After printing, specimens were post-cured using LC-3DPrint Box machine for 30-min. at 60 °C temperature
Formlabs Denture Base Resin LP Formlabs Inc., Somerville, MA, USA	55–75% *w*/*w* urethane dimethacrylate, 15–25% *w*/*w* methacrylate monomers, and <0.9% *w*/*w* phenyl bis(2,4,6-trimethylbenzoyl)-phosphine oxide	Form 2 printer was used to print specimens with 50 µm Printing layer thickness and 0-degree printing orientation. After printing, specimens were post-cured using FormCure machine for 30-min. at 60 °C temperature
Hard Denture Reline GC AMERICA INC, Alsip, IL, USA	Isobutyl methacrylate dibenzoyl peroxide	Powder/liquid ratio is 15 mg powder to 6 mL liquid

**Table 2:** Mean values, SD, and significance of SBS (MPa) between tested groups.

Denture Base Resin and Code		Relining/NPs %			*p*-Value
		0%	2%	4%	
Conventional	Heat-polymerized acrylic resin (HP)	34.9 (11.9) ^a^	32.9 (9.1) MPa	32.5 (16.4)	0.912
Milled	IvoCad (IVO)	43.2 (6.9) ^A^	36.1 (10.1) ^B^	24.9 (8.1) ^A,B^	0.001 *
	AvaDent (AVA)	51.8 (13.3) ^a,b,c,A,B^	36.9 (12.3) ^B^	26.1 (15.9) ^A^	0.003 *
3D printed	FormLabs (FL)	29.7 (9.2) ^b^	22.9 (12.5)	20.0 (9.9)	0.164
	NextDent (ND)	31.1 (11.9) ^c^	22.9 (12.5)	29.7 (15.5)	0.689
		0.000 *	0.033 *	0.372	

* Statistically significant at a *p* < 0.05 level. Lowercase letters indicate significant differences in columns. Capital letters indicate significant differences in rows.

**Table 3:** Two-way ANOVA results.

	Type III Sum of Squares	Df	Mean Square	F	*p*
Intercept	137,622.914	1	137,622.914	975.214	0.000 *
material * concentration	8325.627	14	594.688	4.214	0.000 *
Error	16,934.495	120	141.121		
Total	162,883.037	135			

* Statistically significant at a *p* < 0.05 level^.^

## Data Availability

Data are contained within the article.
